# The Treatment of Diphtheria

**Published:** 1880-02

**Authors:** Rudolph Seiffert


### ARTICLE IX.

*The Treatment of Diphtheria.*

I beg to call the attention of my medical brethren to a mode of treatment of diphtheria which has given me, so far, even in very serious cases, extraordinarily good results, and which is very simple in its application.

My main remedy in the treatment of this disease has been for some time past the inhalation of carbolic acid, diluted, through a sponge. The sponge is saturated with a solution of carb. acid (the strength ranging from 1 part in 200 to 1 part in 100 of water), then placed in a wire holder, formed so as to fit over mouth and nose. This inhaling apparatus is fastened to the patient's mouth and nostrils so that respiration has to take place through the carbolated sponge. I order these inhalations to be repeated every two hours, and each inhalation to extend through about half an hour.

Within twenty-four hours after the beginning of these inhalations the membranes become loose, and are gradually either swallowed or expectorated, and they do not re-appear with the same severity. But if similar formations do take place, they appear thinner and lose that characteristic coloring, growing gradually lighter and more transparent, until they disappear altogether, which is generally within three days, and with this the mucous membrane assumes its normal condition.

As the pain in the throat is ameliorated with every inhalation, the little patients notice this so markedly that every time they experience any pain, they request the application of the carbol. sponge.

In the cases of larger children, more gargling is possible. I order the throat to be gargled with warm chamomile (German) tea, in order to assist in the expectoration of the loose membranes, which procedure, of course, is inadmissible with smaller children.

After every application the sponge ought to be well washed in hot water, and if there be several patients in the same family, each one ought to have its own apparatus, or at least its own sponge. To prevent the carbol. acid sol. from affecting the lips, it is well to oil the lips previous to applying the inhaling apparatus.

The above method of inhalation is very simple and easily applied to children of any age. A few kind words generally suffice to induce them to take to the operation of inhaling with good nature.

Besides the inhalation I use quinine, and in order to keep the bowels open, the fl. ext. of rhamnus frangul. With larger children, I occasionally order gargling with a weak sol. of carb. acid.

As remarked above, this mode of treatment has always given me very satisfactory results. I believe, however, in order to fully establish the merits of this mode of treatment, a larger number of observations is necessary than can be made by a single practitioner. I therefore decided to publish my experience, so as to induce other physicians to test my method.

I wish yet to mention that the apparatus for inhaling, complete with sponge, is kept in stock by Mr. Arend, 179 East Madison street.

DR. RUDOLPH SEIFFERT.

